# Fifty Percent Prevalence of Extracampine Hallucinations in Parkinson’s Disease Patients

**DOI:** 10.3389/fneur.2015.00263

**Published:** 2015-12-21

**Authors:** Ruth A. Wood, Sarah A. Hopkins, Kuven K. Moodley, Dennis Chan

**Affiliations:** ^1^Department of Medicine, Brighton and Sussex Medical School, Brighton, UK; ^2^Department of Medicine for the Elderly, Addenbrooke’s Hospital, Cambridge, UK; ^3^Department of Clinical Neurosciences, University of Cambridge, Cambridge, UK

**Keywords:** Parkinson’s disease, hallucinations, extracampine hallucinations, cortical Lewy body disease, visual hallucinations

## Abstract

Extracampine hallucinations (EH), the sense of a presence or fleeting movement in the absence of an associated visual percept, have been reported in Parkinson’s disease (PD) patients but their prevalence, characteristics, and temporal relationship to visual hallucinations (VH) remain unclear. Given that, VH are predictive of cognitive impairment in PD, improved understanding of EH may have significant prognostic implications. The objective of this study is to evaluate the prevalence and characteristics of EH in a large unselected population with PD and to assess the temporal relationship between EH, VH, and memory decline. Cross-sectional data were collected from 414 PD patients using a questionnaire circulated via an online patient community. Data were obtained regarding the occurrence, timing, and characteristics of VH and EH and symptoms of PD, disease duration, disease severity, and medication history. About 50.4% of respondents reported EH and 15.5% reported VH. EH were typically experienced alongside, rather than behind, the individual (*p* < 0.001) without clear lateralization (*p* = 0.438) and were more likely to be of unfamiliar presences (*p* < 0.001). The occurrence of EH was associated with Hoehn and Yahr score (*p* = 0.002) but not disease duration (*p* = 0.158). EH onset was associated with VH onset (*p* = 0.046) and occurred after the onset of anosmia (*p* < 0.001), cognitive decline (*p* = 0.002), and sleep disturbance (*p* = 0.002). The reported prevalence of EH in PD patients was threefold greater than that of VH, with similar timings of onset, suggesting that EH are under-recognized and under-reported. Further work is needed to determine whether EH are predictive of cognitive decline.

## Introduction

Hallucinations, defined as “*sensory perceptions without external stimulation of the relevant sensory organ*” (1), are a common feature of Parkinson’s disease (PD) and are a poor prognostic marker associated with increased risk of nursing home placement and cognitive decline (2, 3). Various types of hallucinations have been described in PD, including formed visual hallucinations (VH), auditory hallucinations, and extracampine hallucinations (EH), the latter denoting hallucinations occurring outside the visual field (*campus*, from Latin, *field*) (4, 5).

Of these hallucinations, VH are the most commonly described with the lifetime prevalence in PD patients approaching 75% (6). The presence of VH is typically a late feature of PD (4, 7) and is predictive of cortical Lewy body pathology at post-mortem (8, 9). VH in PD are associated with the occurrence of cognitive dysfunction, daytime somnolence, and sleep disorders (4, 10, 11). Although their neural basis is not yet fully established, structural and functional neuroimaging studies have implicated a variety of brain systems, including anterior visual pathways, brainstem, and visual association regions in their pathogenesis (12). More specifically, evidence suggests a role for altered cortical visual processing in the pathogenesis of VH in PD (13).

In addition, antiparkinsonian drug therapy has been reported to precipitate psychosis (14, 15) with certain drugs, particularly dopamine agonists, being linked to VH in PD (16). However, dopaminergic drug use is not sufficient to explain the occurrence of all VH in PD (4). Furthermore, drug-induced psychosis in PD may persist even when the offending medication is withdrawn (17), and there appears to be a poor correlation between drug exposure and symptom severity (18).

In contrast to VH, EH (5) are not associated with any visual percept. Instead, EH are experienced as the sense of a presence beside or behind, or as a fleeting sense of movement alongside, the individual perceiving this phenomenon. In previous studies of hallucinoses associated with PD, EH have been classified as “minor hallucinations,” which, when combined with illusions, is associated with a prevalence ranging from 17 to 72% (4, 19–21). Several explanations exist for this wide range in reported prevalence, including heterogeneity of study populations and methodological differences in assessing the presence of these symptoms (21).

The occurrence of EH is not specific to PD; they have been reported in cerebrovascular disease, epilepsy, sleep paralysis, and traumatic brain injury (22–24). Aside from neurological disorders, EH have been described by individuals in situations associated with extreme physical hardship, such as shipwreck survivors, mountaineers, and polar explorers (25–27), perhaps most famously by Ernest Shackleton during his failed Antarctic expedition of 1914–1917.

… it seemed to me often that we were four, not three. I said nothing to my companions on the point, but afterwards Worsley said to me, ‘Boss, I had the curious feeling on the march that there was another person with us’ (25)

In the context of neurodegenerative diseases, the presence of VH is a relatively specific feature of cortical Lewy body disease (9, 28) and forms one component of the diagnostic criteria for Dementia with Lewy Bodies (DLB) and Parkinson’s Disease Dementia (8, 29). Given that, VH are predictive of future cognitive decline (30–32), it is possible that other types of hallucinosis may also have predictive value, notably those likely to be indicative of cortical involvement.

Extracampine hallucinations are by definition less overt than VH, and therefore may be both under-reported by patients and under-investigated by clinicians, who do not routinely include questions about EH in their history taking (5). Additionally, while the neural substrate of EH has yet to be clarified, their phenomenological nature would implicate higher order association cortices in EH generation beyond any reasonable doubt. As such, it is important to establish whether EH represent a cortical marker of disease in patients with PD, and thus are predictive of future cognitive and clinical decline. Such determination would have major implications for biomedical research, in terms of disease phenotyping and natural history studies, and for clinical practice, with the assessment of EH in patients with PD potentially acting as a prognostic marker alerting clinicians to impend cognitive decline. In practice, EH focused questions could be included in the routine clinical history or, in the future, could be assessed in specialist PD clinics using a validated EH questionnaire similar to those currently in use for the assessment of VH (33).

However, prior to any such studies, the first step is to obtain a better understanding of the occurrence of EH in patients with PD, in view of the limited information available at present. The primary objective of this study was therefore to determine the prevalence of EH in patients with PD. The secondary objectives are to identify the main characteristics of EH (for example, emotional content, familiarity of percept, laterality of EH) and to assess the temporal relationship between the onset of EH and other key symptoms of PD, specifically VH, cognitive impairment, and REM sleep behavior disorders. In order to obtain these data from a more representative sample of PD patients, and thus offset the potential ascertainment bias associated with sampling from a specialist PD clinics, information about EH occurrence were acquired by way of a web-based questionnaire made available to an online social networking community of patients, *patientslikeme*^®^ (34).

## Materials and Methods

### Recruitment and Administration of Questionnaire

*patientslikeme*^®^ is an online patient network where members share personal health data to track their disease progression, communicate with other patients, and contribute to research studies. Members of the community are regularly invited to participate in optional questionnaire-based research studies (34). Although patients with any illness are able to sign up to the website, *patientslikeme*^®^ has a large online community of individuals with PD; at the time of this study, there were 6841 such members.

**Figure 1:**
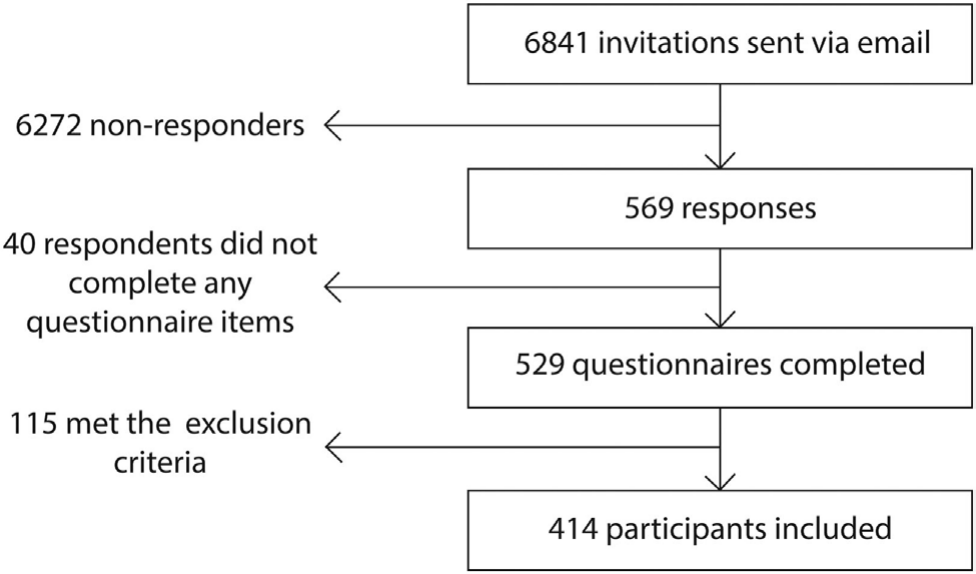
**Flowchart illustrating the recruitment and progression of participants through the study**.

All members of the *patientslikeme*^®^ community who had previously self-identified as having PD were invited to participate in the study via email (Figure 1). The link provided in the email enabled potential participants to access the study information and indicate whether or not they wished to participate. Submission of the completed questionnaire was interpreted as provision of consent for study enrollment in line with ethical guidelines from The University of Sussex on the acquisition of anonymous questionnaire-based data from patient groups (35). The online survey remained open for 1 week and to verify the test–retest reliability of the EH questionnaire and, in line with routine practice in questionnaire-based surveys hosted on the *patientslikeme*^®^ platform, all participants were invited by email to complete a retest phase 2 weeks later in which an identical questionnaire was administered.

### Ethics

Ethical approval for this study was obtained from the South East Coast – Surrey Regional Ethics Committee (reference number 12/LO/1001).

### Inclusion/Exclusion Criteria

Those included in the study were adults with a self-reported diagnosis of PD. Respondents were excluded if they had self-reported Lewy body dementia, PD with associated dementia, a non-PD neurodegenerative disorder, or moderate-to-severe visual impairment (cataracts, macular degeneration, diabetic eye disease, or registered blind).

### Questionnaire

The study instrument was a novel, web-based, questionnaire, which was completed via the *patientslikeme*^®^ online platform. The questions were generated by the study authors with input from patients with PD and their caregivers. Participants were informed of the nature of the study in terms of the information being requested but were blinded to the specific hypotheses being tested, in order to eliminate reporting bias. In order to increase the response rate, an email reminder was sent after the initial launch.

All data were self-reported and information was gathered regarding demographics, co-morbidities, and current and previous experiences of illusions (*Q1*, Table 1), VH (*Q22–26*), and EH (*Q2–21*). Two questions were used to assess whether or not participants had experienced EH (*Q2 and Q6*) and a participant was defined as having had an EH if they answered yes to one or both of these questions.

**Table 1:** **Questionnaire items as posed to participants**.

Modified Hoehn and Yahr score	Parkinson’s disease progresses through five stages, called “Hoehn and Yahr” stages. Please indicate which of the five statements below best describes your status at the moment
	1. I have symptoms on one side of my body only
	2. I have symptoms on both sides of my body but no problems with balance or walking
	3. I’m having problems with my balance and walking
	4. I can still stand and walk but with great difficulty
	5. I cannot stand or walk independently; I need help or a wheelchair
Illusions	Q1. Have you ever looked at something and thought it was something else altogether? (*For example: spots on the wall looking like insects or a lampshade cover being mistaken for a person*.) *Yes/No*
EH: feeling of a *movement*
	Q2. Have you ever experienced a feeling of movement past you when there was nothing there to account for this feeling? (*Some patients report a sensation that something has flashed past them, out of the corner of their eyes*.) *Yes/No*
	Q3. Where was the feeling of movement in relation to you? *Front/Behind/To the side*
	Q4. If to the side, do they occur more on the left or the right? *Mostly on the left/Mostly on the right/There is no pattern/I have not noticed a pattern/Does not apply*
	Q5. Are these experiences more common at a particular time of day? *Morning/Afternoon/Evening/Night/There is no pattern*
EH: feeling of a *presence*
	Q6. Have you ever felt of imagined a presence either behind or alongside you when there was nothing there to account for this feeling? *Yes/No*
	Q7. Do you feel as though this presence is human, animal or other? *Human/Animal/Other*
	Q8. Is the presence familiar to you? *Yes/No*
	Q9. Does the presence ever speak to you or make another noise? *Yes/No*
	Q10. Does the presence ever touch you? *Yes/No*
	Q11. Are these experiences always the same? *Yes/No*
	Q12. Have any of your medications ever made these experiences better OR worse? *Yes/No*
	Q13. If you have ever taken any of the following medications* please indicate how they affected these experiences. *Improved symptoms/Worsened symptoms/No effect on my symptoms/I have never taken this medication*
	Q14. If you have ever taken any other medications for your Parkinson’s disease, please indicate what these were and how they affected these experiences
	Q15. Do you have a reduced sense of smell? *Yes/No*
	Q16. Have you noticed a decline in your memory or thinking? *Yes/No*
	Q17. Have you noticed a change in your ability to walk or maintain balance? *Yes/No*
	Q18. Have you noticed that your sleeping has become more restless or disturbed? *Yes/No*
	**For questions 15–18, if the answer was yes participants were also asked**
	Q19. Did you notice this before or after you FIRST noticed the presence? *Before/After*
	Q20. Are you able to estimate how much time passed between noticing this and first becoming aware of a presence? *Yes, I can/No, I cannot*
	Q21. If yes, please estimate this period (in days, weeks, months, or years)
VH
	Q22. Have you ever SEEN things that were not really present or that other people could not see? (*For example, some patients report seeing animals or children that others cannot see*.) *Yes/No*
	Q23. If you answered YES, when did this occur in relation to feeling/imaging the sense of a presence? *Before/After/They occurred at the same time/I am not sure which occurred first*
	Q24. If you answered BEFORE or AFTER please estimate this period (in days, weeks, months or years)
	Q25. How often do you see things that are not really present or that other people cannot see? *Less than once a week/At least once a week/Several times a week*
	Q26. What do you generally see during these episodes? *The images are not fully formed/I see adult beings/I see children/I see animals or insects/I see shadows that may sometimes move/I see other everyday objects*
	**For each type of hallucination (EH and VH) the following questions were asked**
	Q27. When was the last time this happened? *Within the last week/Within the last month/Within the last 3 months/Within the last 6 months/More than 6 months ago*
	Q28. These experiences are: *Pleasant or reassuring/Unpleasant or frightening/Neither*
	Q29. These experiences make me feel: *Happy/Sad/Neither*
Sleep
	Q30. I sometimes have very vivid dreams *Yes/No*
	Q31. I have been told that I kick and/or punch in my sleep *Yes/No*
	Q32. I have been told that I shout, swear or laugh in my sleep *Yes/No*

*In addition to the potential responses listed, participants were also able to skip the question*.

*Co-careldopa, Co-benyldopa, Stalevo, Rotigotine, Pramipexole, Ropinirole, Benzotropine, Trihexyphenidyl, Selegiline, Rasagiline, Entacapone, Amantadine, Rivastigmine, Donepezil, Galantamine.

Participants were asked to characterize EH in terms of EH location relative to themselves (*Q3*), laterality (*Q4*), emotional content (*Q28*), time of day of occurrence (*Q5*), form (*Q7*), and familiarity (*Q8*). They were also asked about any auditory or tactile (*Q9–10*) components, whether their EH were stereotyped (*Q11*) and the effect of medications on their EH, in terms of aggravation or amelioration (*Q12–14*). All known trade names for PD medications were used, to help ensure correct identification of medications used by participants from different countries.

Data were also collected regarding the relationship between the onset of EH and other features of disease, such as VH, memory decline, anosmia, and sleep disturbance. Disease severity was assessed using a modified version of the Hoehn and Yahr score, already in use by *patientslikeme*^®^, in which patients identify their disease severity (Table 1).

### Statistical Analysis

Statistical analysis was performed using IBM SPSS Statistics Version 22.0. Descriptive statistics were used to analyze the characteristics of EH in terms of laterality (left/right), familiarity (familiar/unfamiliar), emotional content (pleasant/unpleasant/neither), associated emotional response (happy/sad/neither), and time of day (morning/afternoon/evening/night). Chi-squared testing was undertaken to determine whether there were significant differences between the expected and observed frequencies of these variables.

Respondents were divided into two groups, those with EH and those without, and the distribution of variables, such as age, gender, disease duration, and severity and presence of VH within these groups were compared using an independent *t*-test, Mann–Whitney *U*, or a chi-squared test (with or without trend) depending on whether the variable being analyzed was continuous or categorical and normally distributed. Normality of data was assessed using visual inspection of histograms, QQ plots and box plots, and Shapiro–Wilk testing.

A logistic regression analysis was undertaken to determine if a set of pre-specified variables (age, gender, disease duration, disease severity as specified by Hoehn and Yahr score, and presence of VH) were predictive of the occurrence of EH (36). These potential predictor variables were selected based on prior clinical knowledge and pre-specified hypotheses, explicitly that the prevalence of EH would not be influenced by demographic factors, such as age and sex, but would be affected by disease duration, disease severity, and VH, putative correlates of accumulating cortical Lewy Body pathology. A Box–Tidwell test was used to ensure there was linearity for continuous and transformed categorical variables. The dependent variable, the occurrence of EH, was dichotomous and assigned a value of 1 (EH occurrence) or 0 (no EH occurrence).

## Results

### Recruitment

Data from 414 respondents were included in the study. Of the 6841 individuals with PD who were invited to participate, 569 responded indicating a response rate of 8.3%. Forty (9.7%) respondents were excluded as they had not completed the questionnaire, and 115 (20.2%) were excluded on the basis of the exclusion criteria outlined above.

### Participant Demographics

The mean age of respondents was 61.9 years (standard deviation 8.2 years, range 33–88 years) and 48.2% (199/413) of the cohort were female. About 83.5% (324/388) of participants were resident in the United States or United Kingdom with others living in Europe, Canada, Australasia, South America, and the Middle East. The mean Hoehn and Yahr score was 2.11 (SD 0.99, range 1–5) with 94.9% (391/412) of participants reporting a score between 1 and 3. Mean disease duration was 7.3 years (SD 5.0 years, range 1–29 years).

### Prevalence of Hallucinations

**Figure 2:**
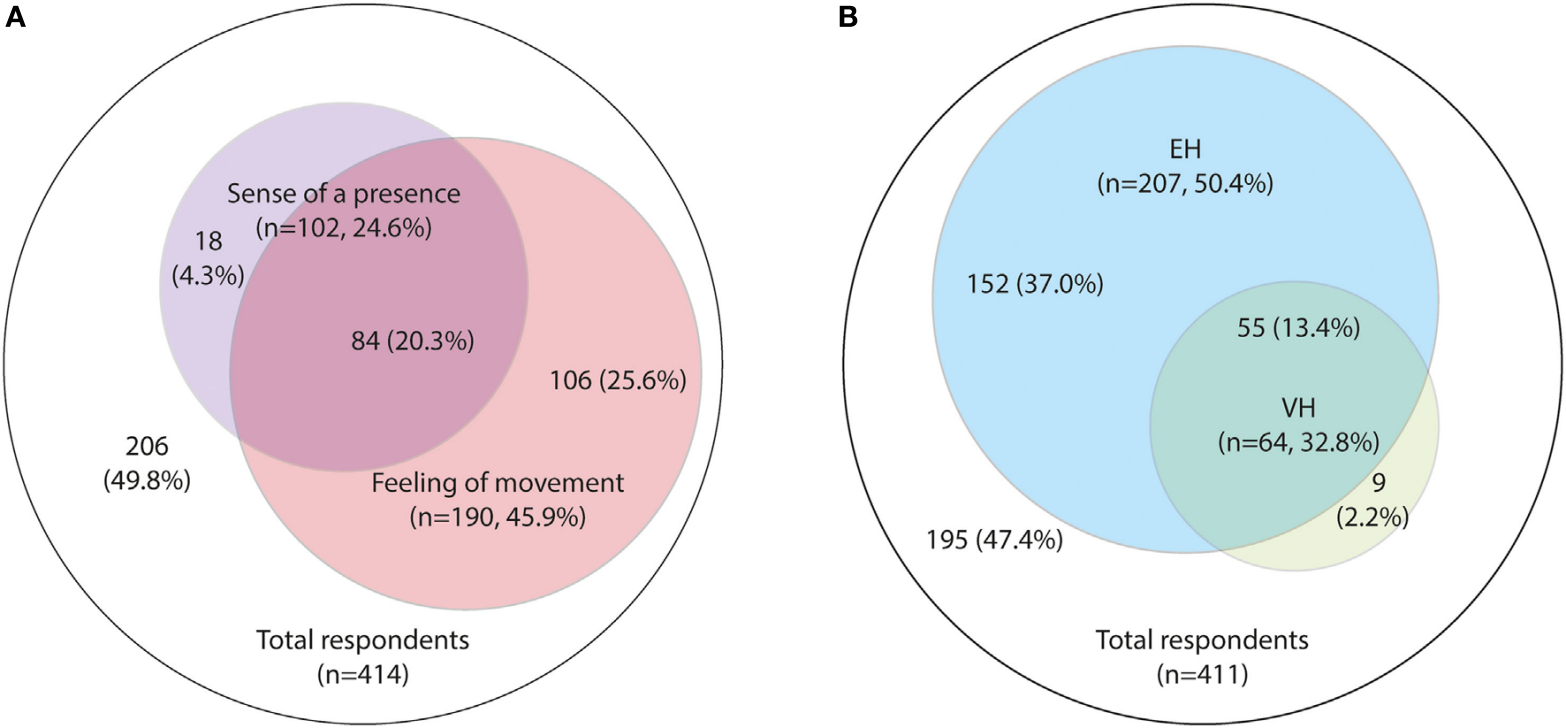
**Prevalence of hallucinations**. **(A)** Extracampine hallucinations (EH), “feeling or imagining a presence” versus a “feeling of movement”; **(B)** Prevalence of EH and visual hallucinations (VH).

About 33.9% (140/413) of respondents reported illusions and 15.5% (64/412) had experienced VH. More than half of respondents reported EH; 45.9% (190/414) reported a “feeling of movement” passing them and 24.6% (102/414) reported “feeling or imaging a presence that was not truly there.” Given that, 20.3% reported both symptoms, 50.4% (208/413) experienced either a “feeling of movement” or had “felt or imagined a presence” (Figure 2).

### Predictors of EH

Neither age nor gender was predictors for the occurrence of EH (Table 2). Mean disease duration did not differ significantly between the EH and non-EH groups (*p* = 0.158), but those with EH were more likely to have a higher Hoehn and Yahr Stage (*p* = 0.002) (Table 2).

**Table 2:** **A comparison of participants with and without extracampine hallucinations (EH)**.

	Participants with EH	Participants without EH	*p*-Value
Number of respondents	208 (50.4)	204 (49.6)	–
Female respondents (%)	107 (54.0)	91 (46.0)	0.165*
Age (years) (SD)	62.1 (8.1)	61.6 (8.3)	0.520^†^
Visual hallucinations reported (%)	55/207 (26.6)	9/204 (4.4)	<0.001*
Disease duration (years) (SD)	7.5 (5.0)	7.1 (4.9)	0.158^‡^
Hoehn and Yahr Stage (%)			**0.002^§^**
Stage 1	67 (32.2)	87 (42.9)	
Stage 2	35 (16.8)	47 (23.2)	
Stage 3	96 (46.2)	59 (29.1)	
Stage 4	10 (4.8)	8 (3.9)	
Stage 5	0 (0)	2 (1.0)	

*Chi squared test.

*^†^Independent *T*-test*.

*^‡^Mann–Whitney *U**.

*^§^Chi squared test with trend (Hoehn and Yahr classes collapsed to Stage 1, Stage 2, and Stage ≥ 3)*.

*Bold font denotes statistically significant results with a cut off of p < 0.05*.

### Characteristics of EH

Extracampine hallucinations were more likely to occur alongside rather than directly in front of or behind the person experiencing them (*p* < 0.001) (Table 3). Although EH did lateralize to the left or right in 47.0% (78/166) of cases this was a non-significant finding (*p* = 0.438) with a similar proportion of respondents (53.0%, 88/166) stating either that there was no left/right pattern or that they had not noticed such a pattern. Of the 47.0% (78/166) that did lateralize more were reported on the right side but this did not reach statistical significance (*p* = 0.070).

**Table 3:** **Characteristics of reported extracampine hallucinations (EH)**.

Feature		Observed result (%)	*p*-Value*
Laterality	Front	11 (6.4)	**<0.001**
	Behind	7 (4.1)	
	Side	153 (89.5)	
	No laterality	88 (53.0)	0.438
	Left/right preference	78 (47.0)	
	Left^†^	31(39.7)^†^	**0.007**^†^
	Right^†^	47 (60.3)^†^	
Familiarity	Familiar	34 (34.0)	**<0.001**
	Unfamiliar	66 (66.0)	
Emotional content	For “a feeling of movement”		
	Pleasant	3 (1.6)	**<0.001**
	Unpleasant	33 (17.5)	
	Neither pleasant nor unpleasant	153 (81.0)	
	For “the sense of a presence”		
	Pleasant	10 (9.8)	**<0.001**
	Unpleasant	24 (23.5)	
	Neither pleasant nor unpleasant	68 (66.7)	
	Happy	2 (1.1)	**<0.001**
	Sad	7 (3.7)	
	Neither	180 (95.2)	
EH content	Animals	20 (20.2)	**<0.001**
	People	70 (70.7)	
	Inanimate objects	9 (9.1)	
Other sensory components	Auditory component	6 (5.9)	**<0.001**
	No auditory component	95 (94.1)	
	Tactile component	11 (10.9)	**<0.001**
	No tactile component	90 (89.1)	
Stereotyped	Always the same	29 (29.6)	**<0.001**
	Not always the same	69 (72.6)	
Timing	Occur at a particular time of day (*morning/afternoon/evening/night*)	49 (26.2)	**<0.001**
	Occur at any time of day or night	138 (73.8)	

*Chi-squared testing.

*^†^Subgroup analysis*.

*Bold font denotes statistically significant results with a cut off of p < 0.05*.

Extracampine hallucinations were more likely to feel “unfamiliar” than “familiar” (*p* = 0.001) and seemed to be emotional neutral experiences making people feel neither happy nor sad (*p* < 0.001), and neither pleasant nor unpleasant, whether they had reported “a feeling of movement” (*p* < 0.001) or the “sense of a presence” (*p* < 0.001) (Table 3).

### Timing of EH

In those who reported EH these were often recently experienced; 81.6% (151/185) of those who reported a “feeling of a movement passing them” and 72.3% (73/101) of those who reported a “felt or imagined presence” had experienced this phenomenon within the previous three months. EH were not associated with a particular time of day (morning/afternoon/evening/night) (*p* < 0.001).

### Relationship Between EH and Other Symptoms Associated with PD

Extracampine hallucinations co-existed with other PD symptoms. Of those who reported EH, 75.5% (77/102) also had anosmia, 73.3% (74/101) reported a decline in their memory or thinking, 72.5% (74/102) had restless sleep, and 77.4% (27/35) had experienced a change in their balance or walking.

The onset of anosmia, memory decline, and sleep disturbance were all more likely to precede the onset of EH in our cohort (*p* < 0.001, *p* = 0.002, and *p* = 0.002, respectively), but there was no clear temporal relationship between the onset of EH and the onset of motor symptoms (*p* = 0.221) in line with the above finding of a lack of association between EH and disease duration (Table 4).

**Table 4:** **Relationships between (A) the onset of EH and other PD symptoms, (B) EH and potential predictor variables, and (C) EH and medication**.

**(A) Temporal relationships between the onset of EH and other PD symptoms**						

**Symptom**	**Before EH (%)**	**After EH (%)**		**Coincides with EH (%)**		*p*-Value*

VH	9 (22.0)	8 (26.8)		9 (51.2)		**0.046**
Anosmia	57 (86.4)	9 (13.6)		^†^		**<0.001**
Memory decline	44 (69.8)	19 (30.2)		^†^		**0.002**
Sleep disturbance	44 (69.8)	19 (30.2)		^†^		**0.002**
Motor symptoms	15 (62.5)	9 (37.5)		^†^		0.221
**(B) Predictors of the occurrence of EH on a logistic regression analysis**						

**Variable**	**Coefficient**	**SE**	**Odds ratio**	**95% Confidence limits**		***p*-Value**

Age	0.006	0.013	0.994	0.981	1.032	0.644
Sex	0.213	0.212	1.273	0.817	1.873	0.315
Disease duration	−0.002	0.023	0.927	0.955	1.043	0.927
H&Y score	0.162	0.114	1.176	0.941	1.470	0.154
VH	1.958	0.380	7.083	0.067	0.297	**<0.001**
**(C) Medication effect on EH (data from 26/95 participants whose medication affected EH)**						

**Medication**	**Number of responders**	**EH better (%)**	**EH worse (%)**	**No effect (%)**		*p*-Value*

Co-careldopa	24	8 (33)	5 (21)	11 (46)		0.325
Rotigotine	7	0 (0)	4 (57)	3 (42)		0.160
Pramipexole	17	1 (6)	13 (76)	3 (18)		**0.010**
Ropinirole	18	2 (11)	9 (50)	7 (39)		0.229
Selegiline	7	0 (0)	2 (29)	5 (71)		0.066
Rasagiline	12	0 (0)	1 (8)	11 (92)		**<0.001**
Amantadine	12	3 (25)	4 (33)	5 (42)		0.779

*EH, extracampine hallucinations; VH, visual hallucinations*.

*Chi squared testing.

*^†^Data not requested on the questionnaire*.

*Bold font denotes statistically significant results with a cut off of p < 0.05*.

### Relationship Between EH and VH

In 52.8% (19/36) of reported cases, EH onset was described as occurring around the same time as VH, with 25% (9/36) and 22.2% (8/36), respectively, reporting EH onset before and after VH onset. This finding reached statistical significance (*p* = 0.046) (Table 4).

A major overlap was observed between those reporting VH and those reporting EH (Figure 2); 85.9% of those with VH also had EH. Those who reported EH were significantly more likely to report VH (26.6%, 55/207) than those who did not report EH (4.4%, 9/204) (*p* < 0.001) (Table 4). About 37.0% (152/411) of all respondents reported EH but no VH, and 2.2% (9/411) reported VH but no EH (Figure 2).

### Predictors of EH

A binary logistic regression was performed to ascertain the effects of age, gender, disease duration, disease severity (Hoehn and Yahr score), and the presence of VH on the likelihood that the participants had experienced EH. The logistic regression model was statistically significant [χ^2^(5) = 44.463, *p* < 0.001] and explained 13.9% (Nagelkerke *R*^2^) of the variance in the presence of EH. Overall, it correctly predicted the occurrence or absence of EH in 62.4% of respondents; it correctly classified 37.9% of those experiencing EH and 87.1% of those not experiencing EH. Of these variables, only the presence of VH was found to be significantly predictive of EH occurrence (Table 4).

### Medication

Participants who had experienced EH were asked if their medications made their EH better or worse. Overall medications did not appear to have an effect upon EH (*p* < 0.001) with 72.6% (69/95) of respondents stating they had not noticed an effect.

27.4% (26/95) reported that their medication had affected their EH and provided further data regarding individual drugs (Table 4). The following data were collected only from these 26 participants therefore the number of responses for each drug was small. For some drugs (co-benyldopa, stalevo, benzotropine, trihexyphenidyl, entacapone, rivastigmine, donepezil, and galantamine), information was obtained from fewer than five respondents, and these small sample sizes precluded meaningful interpretation; for example, only three patients in this subgroup were taking rivastigmine. Of the remaining drugs, 13/17 respondents stated that pramipexole significantly worsened EH (*p* = 0.010). No significant drug effect on EH was noted for other drugs (Table 4).

### Test–Retest Reproducibility of Responses

52.9% (301/569) of participants completed a retest phase in which they retook an identical questionnaire. Analysis of this data by *patientslikeme*^®^ indicated that our questionnaire had a high test–retest reliability (Cronbachs α = 0.94).

## Discussion

The prevalence of EH in patients with PD, their characteristics and association with other key clinical features of PD were investigated using a web-based questionnaire circulated to the online patient community *patientslikeme*^®^. To the best of our knowledge, this is the first large cross-sectional study to focus on the occurrence of EH in patients with PD. The main finding of the study was that EH were reported by over 50% of respondents, representing a prevalence more than three times greater than that of VH (50.4 versus 15.5%). Although there is no prior study specifically exploring the prevalence of EH in PD against which these study results can be compared, this 50.4% prevalence figure falls within the range of reported prevalences of minor hallucinations in PD (4, 19–21), which includes EH.

Extracampine hallucinations were significantly more likely to be experienced as a presence alongside, rather than behind, the individual (*p* < 0.001), to be an unfamiliar rather than a familiar presence (*p* < 0.001), for the presence to be that of a person rather than an animal or inanimate object (*p* < 0.001), and to be emotionally neutral in content (*p* < 0.001). EH did not tend to be stereotypical in nature (*p* < 0.001), and did not tend to have an auditory or tactile component (*p* < 0.001). There was no significant association between occurrence of EH and time of day (*p* < 0.001).

With regard to other aspects of PD, there was a statistically significant (*p* = 0.002) association between EH occurrence and disease severity, as measured by the Hoehn and Yahr stage, but not with respondent age, gender, and disease duration of PD. The presence of VH was predictive of EH and the majority of respondents who reported EH also described VH (85.9%). In over 50% of respondents, EH and VH onset occurred around the same time.

Surprisingly, and in contrast to previous studies reporting that the onset of VH was predictive of future cognitive decline, in this study respondents stated that EH occurred after the onset of cognitive problems, as well as after anosmia and sleep disturbance. This was an unexpected finding. One possibility is that these results may be skewed by the greater difficulty in recognizing the onset of EH, given its current under-reporting and under-recognition. However, another possibility is that the Lewy body pathology may affect the cortical systems underpinning EH later than those involved in cognition. That said, this would seem a less plausible explanation when the neural substrate of EH is considered. While this is yet to be established, given that EH represent percepts that are by definition devoid of any visual form, it is probable that they are of cortical origin rather than indicative of a disorder of primary sensory pathways. This is reinforced by a recent study investigating the imaging changes associated with the “sense of a presence,” both in a small cohort of patients with neurological disorders (epilepsy, stroke but no PD patients) and healthy subjects in whom this phenomenon was induced artificially using synchronous and asynchronous stimulation (37). The brain regions most commonly associated with “the sense of a presence” were the frontoparietal cortex, insular cortex, and temporoparietal cortex. With regard to the latter, the clinicopathological observation that VH in Lewy body disease were strongly associated with a high density of Lewy bodies in the temporal lobe (9) may provide a clue as to the cortical substrate of EH. Given that, the onset of VH commonly precedes that of cognitive impairment in PD, the expectation might have been that the latter would also be preceded by the occurrence of EH. This will need to be clarified by way of future prospective natural history studies of EH in PD patients without cognitive impairment.

Insufficient data were generated to draw any firm conclusions regarding the effect of medications on EH. Although the majority of patients reported that their medications did not influence their EH, a minority did notice an effect with the only significant finding being that pramipexole worsened EH.

This study has limitations. The use of a web-based questionnaire to obtain responses from an online community of patients not diagnosed, or under the care of, the study authors means that there is no mechanism to confirm the diagnosis of PD or the Hoehn and Yahr score. Knowledge is also lacking on the demographic details of the respondent group and given the online nature of both the community and the questionnaire, it is possible that the respondents as group may not reflect the entire socio-economic breadth of the wider PD patient population. Additionally, this study is potentially vulnerable to the biases associated with all questionnaire-based research studies (selection, recall, and responder bias). With regard to disease severity of the study respondents, the mean Hoehn and Yahr score was 2.1, which may reflect the fact that patients with more severe disease could find completing online questionnaires more challenging. Finally, in order to ensure some degree of homogeneity among the test population, this study did not obtain responses from patients with PD dementia (PDD) and DLB, and no data are currently available regarding the prevalence of EH in the general population.

Future research will aim to determine the prognostic significance of EH, notably with regard to predicting later cognitive decline, as well as to establish the prevalence of EH in patients with PDD and Lewy body dementia.

## Conclusion

The occurrence of EH in 50.4% of respondents suggests that EH is an under-reported and under-recognized symptom of PD. Given that, the occurrence of VH represents one of the core criteria for the diagnosis both of PDD and DLB, identification of EH may be of high clinical significance The likely neural substrate of this phenomenon suggests that EH may indicate cortical involvement in PD and thus be predictive of future cognitive decline.

## Conflict of Interest Statement

The authors declare that the research was conducted in the absence of any commercial or financial relationships that could be construed as a potential conflict of interest.
